# DNA amplification with *in situ* nucleoside to dNTP synthesis, using a single recombinant cell lysate of *E. coli*

**DOI:** 10.1038/s41598-019-51917-z

**Published:** 2019-10-30

**Authors:** Thomas D. Loan, Christopher J. Easton, Apostolos Alissandratos

**Affiliations:** 1 Research School of Chemistry, Australian National University, Canberra, Australia; 2 CSIRO Synthetic Biology Future Science Platform, Australian National University, Canberra, Australia

**Keywords:** Biotechnology, Chemical biology, Synthetic biology

## Abstract

Nucleic acid amplification (NAA) is a cornerstone of modern molecular and synthetic biology. Routine application by non-specialists, however, is hampered by difficulties with storing and handling the requisite labile and expensive reagents, such as deoxynucleoside triphosphates (dNTPs) and polymerases, and the complexity of protocols for their use. Here, a recombinant *E. coli* extract is reported that provides all the enzymes to support high-fidelity DNA amplification, and with labile dNTPs generated *in situ* from cheap and stable deoxynucleosides. Importantly, this is obtained from a single, engineered cell strain, through minimal processing, as a lysate capable of replacing the cold-stored commercial reagents in a typical PCR. This inexpensive preparation is highly active, as 1 L of bacterial culture is enough to supply ~10^6^ NAA reactions. Lyophilized lysate can be used after a single-step reconstitution, resulting overall in a greatly simplified workflow and a promising synthetic biology tool, in particular for applications such as diagnostics.

## Introduction

Enzyme-catalyzed nucleic acid amplification (NAA) through the polymerase chain reaction (PCR) has become an essential tool for molecular and synthetic biology^1,2^. Nonetheless, despite great technological advances, more routine use of this powerful tool is still hampered by test affordability and complexity^3–8^. This is of particular interest for important point-of-need applications, such as with diagnostics for health and biosecurity performed by non-specialists in remote settings, where new PCR-based tests have extraordinary potential for the detection of many organisms^5,9^. An important limitation is that the expensive requisite biochemical reagents, primarily deoxyribonucleoside triphosphates (dNTPs) and polymerase enzymes, are produced through separate pipelines encompassing labor-intensive purification strategies^3,10–12^. The purified components require cold storage and skilled handling for application. Therefore, it is evident that routine NAA in the field or within low-tech settings requires further development of simple, streamlined protocols that employ robust and inexpensive reagents.

As dNTPs are the monomers for DNA polymerization, they are essential for any NAA reaction, and are employed in excess to other reagents^2,13^. Frozen stocks require careful handling, as repeat freeze-thaw cycles lead to decomposition and failed applications. To circumvent this problem, we recently showed that lysate of *E. coli* is able to catalyze the enzymatic conversion of dNMPs to dNTP mixtures, suitable for use in PCR^14^. ATP is required in only catalytic amounts as it may be recycled with acetyl phosphate, which is prepared readily from low-cost reagents^14,15^. Nevertheless, though monophosphates do not contain the labile γ-phosphate, they were still synthesized and stored independently, while the PCR was carried out as a separate reaction using commercial DNA polymerase. Bhadra *et al*.^3^ recently showed that polymerases for important NAA applications could be supplied in the form of lyophilized whole-cell biocatalysts (cellular reagents) without loss of sensitivity. This shows that laborious and expensive enzyme purification is not necessary, however it does not address other aspects of NAA complexity, or the possibility of using unphosphorylated deoxynucleosides in preference to dNMPs.

Accordingly, herein we report the development of a recombinant cell lysate of *E. coli* that provides a simplified NAA protocol at very low cost. We show that it is possible to produce dNTP mixtures from cheap and room-temperature stable unphosphorylated deoxynucleosides, using a lysate recombinantly enriched with the deoxynucleoside kinase (dNK) from *Drosophila melanogaster*^16^ that processes all natural deoxynucleosides. The lysate is obtained through minimal processing from a single bacterial culture, and can be employed as part of the PCR mixture to catalyze one-pot transformation of deoxynucleosides to dNTPs for subsequent DNA synthesis by commercial polymerase enzyme, with no reagent transfer or sample handling between reactions. Emboldened by this, the dNK was co-expressed with a high-fidelity DNA polymerase from *Pyrococcus furiosus*^17,18^, resulting in a single lysate able to catalyze the deoxynucleosides to dNTPs synthesis cascades (14 enzymatic steps) as well as DNA synthesis, without addition of any commercial PCR components. Lyophilization has been employed successfully for the storage and stabilization of cell lysates for cell-free protein synthesis and diagnostics^8,19–21^. Analogously, our lysate retains full activity when lyophilized with the cheap disaccharide lyoprotectant sucrose, for use through simple reconstitution in buffer. This cell-free synthetic biology approach affords high lysate activity, as just 1 L of bacterial culture generates enough material to carry out ~10^6^ PCR tests. In this way, costly and labile commercial PCR components are replaced by an affordable, robust preparation, greatly simplifying application. This first example of lysate-catalyzed DNA synthesis from deoxynucleosides further demonstrates the broad utility of cell-free synthetic biology and holds great promise for NAA applications, particularly in low-tech and remote settings where straight-forward protocols are essential.

## Results and Discussion

**Figure 1:**
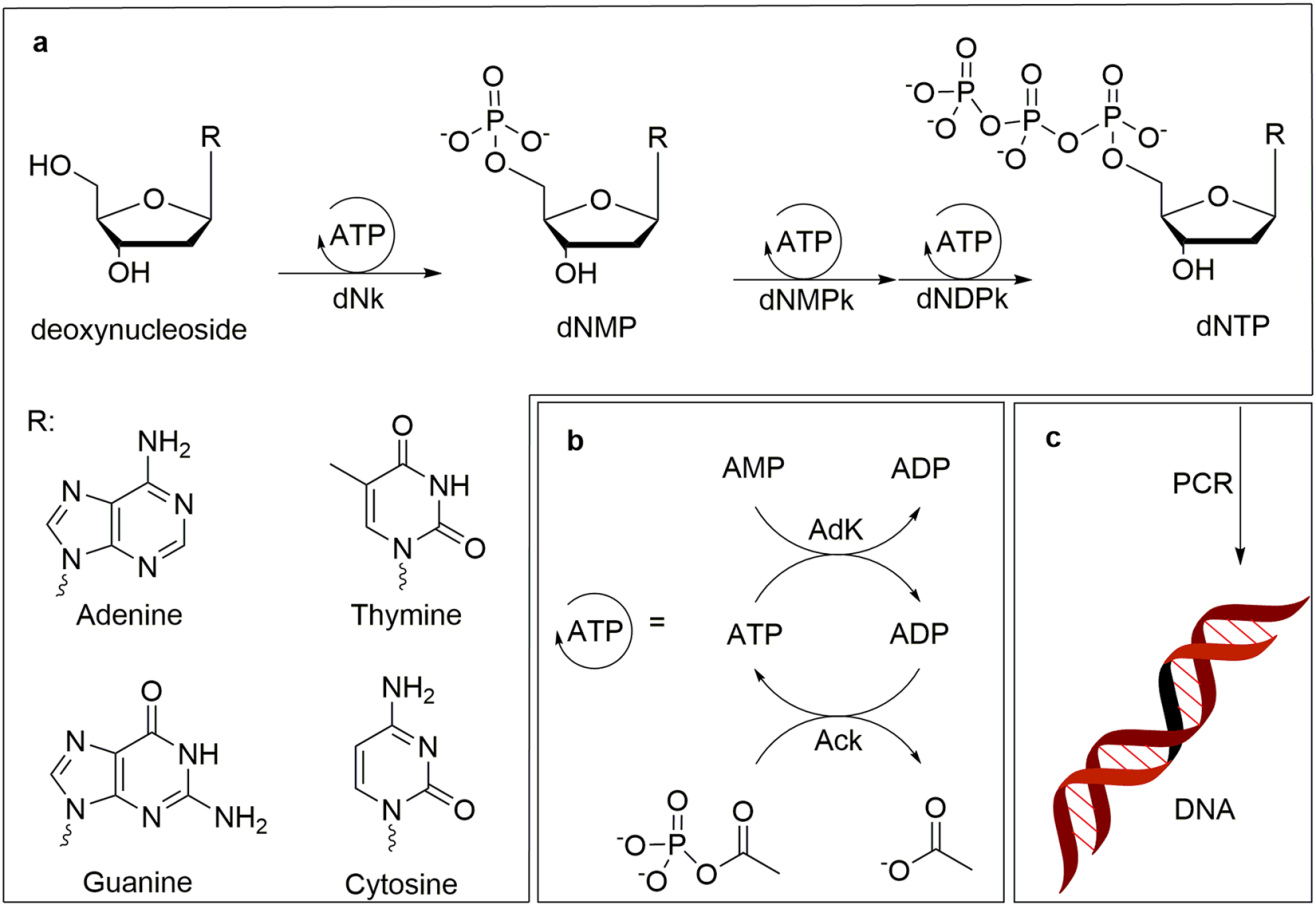
Coupled synthesis of dNTPs and DNA. (**a**) Deoxynucleosides are transformed to dNMPs by recombinant deoxynucleoside kinase (dNK) and further converted to dNTPs by endogenous lysate kinases (dNMPK, dNDPK). (**b**) ATP is recycled by endogenous acetate kinase (AcK) (and adenylate kinase (Adk) to convert any AMP) with acetyl phopshate. (**c**) dNTPs are used directly for DNA synthesis catalyzed by DNA polymerase (PCR). The figure was created using ChemDraw Professional 17.0 (PerkinElmer Informatics).

Initially, the synthesis of dNTPs from the unphosphorylated deoxynucleosides (Fig. 1a) was attempted with native *E. coli* BL21(DE3) lysate. The application of cell lysates for multi-enzymatic cascades has gained increasing popularity due to its potential for efficient and inexpensive production of useful bio-chemicals^22–27^. Previously, we have employed this approach to produce nucleotides from cheap starting materials in quantitative yields with endogenous cofactor recycling driving multiple ATP-dependent steps (>10^2^ turnovers)^26,28,29^. Even though the only dNK known to be present in *E. coli* is thymidine kinase^30^, we have observed that kinase promiscuity in lysates may accommodate non-substrate nucleotide to catalyze the initial phosphorylations^14^. However, in this case, none of the natural deoxynucleosides was converted to a dNMP, indicating that all four necessary activities are absent in the native lysate.

We next considered introducing these activities recombinantly through a modular, cell-free metabolic engineering approach^31^. We envisaged that a lysate enriched with requisite recombinant dNK activities would enable dNMP production and initiate the cascade to dNTPs (Fig. 1a,b). For this, the enzyme from *Drosophila melanogaster* (EC 2.7.1.145) was identified^16^ as it is reported to accept all four deoxynucleosides^16^, thus obviating the requirement for multiple recombinant genes which could impact cell fitness. The codon optimized gene was sub-cloned into the pETMCSIII vector^32^ for expression with an N-(His)_6_-tag. Overnight expression in *E. coli* BL21(DE3) grown in lysogeny broth supplemented with ampicillin (LB-Amp) resulted in high yields of recombinant protein. Ni(II)-affinity purified protein showed monophosphate forming activity in assays with deoxyadenosine (dAdo), deoxycytidine (dCyt), deoxyguanosine (dGuo) and thymidine (dThy) (Supplementary Information, Fig. S1).

**Figure 2:**
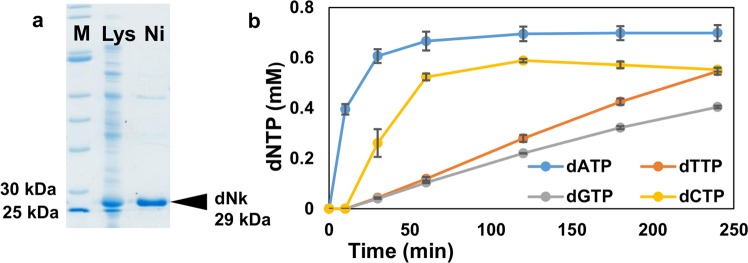
Analysis and application of recombinant cell lysate from the overexpression of dNK. (**a**) SDS-PAGE analysis of lysate from *E. coli* BL21(DE3) overexpressing dNK (Lys), Ni(II)-affinity purified lysate fraction (Ni), and molecular weight marker (M) (NEB Broad Range 10–200 kDa). (**b**) Reaction progress for dNTP synthesis from deoxynucleosides (1 mM each) catalyzed by this lysate. Error bars are the standard deviation of triplicate experiments.

SDS-PAGE analysis of the crude lysate prepared from the engineered *E. coli* (50 mL, LB-Amp) showed the presence of an over-expressed protein of the size expected for dNK (Fig. 2a). The same size protein was also present in the Ni(II)-affinity purified fraction, indicating the protein is N-(His)_6_-tagged. Therefore, recombinant lysate activity for the full conversion of deoxynucleosides through to the dNTPs was evaluated. This depends on active endogenous kinases in addition to the recombinant dNK, for the catalysis of all four three-step cascades as well as the ATP recycling (Fig. 1a,b). When an equimolar mixture of the four deoxynucleosides (1 mM) was incubated with lysate (0.4% v/v), ATP (1 mM) and acetyl phosphate (50 mM), simultaneous production of all dNTPs was observed (Fig. 2b), through monitoring by reversed phase ion-pair (tetrabutylammonium phosphate) HPLC with detection at 259 nm, and by comparison with standardized response curves of commercial samples for quantification (details are provided in the Methods section “Synthesis of dNTPs from Deoxynucleosides and HPLC Analysis”). The yields of the dNTPs produced in this reaction were highly reproducible. In triplicate experiments and based on each deoxynucleoside substrate, after 4 h they were 70 ± 3% for dATP, 55 ± 1% for dCTP, 55 ± 1% for TTP and 41 ± 1% for GTP. Presumably, the different yields and rates of production of the four dNTPs reflect the kinetic parameters of the kinases involved for each of the deoxynucleoside substrates and intermediates (dNMPs, dNDPs). Previous experience indicated that the dNTP concentrations reached here after 4 h would be sufficient to support PCR^14^, so further optimization of dNTP synthesis was deemed unnecessary.

**Figure 3:**
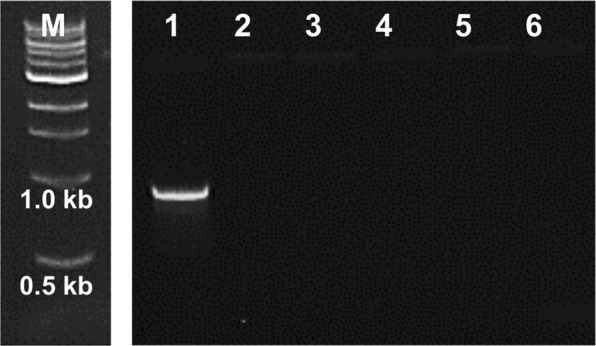
Agarose gel of PCR products using dNTPs synthesized by dNK lysate. NEB 1 kb DNA size marker (M), 25 μL PCR with 2.5 μL dNTP synthesis reaction mixture in place of commercial dNTPs (1), control without DNA template (2), control without deoxynucleosides (3), control without ATP (lane 4), control without acetyl phosphate (5), and control without lysate (6). Expected product size is 0.9 kb. All lanes are from a single gel image, cropped for clarity and conciseness. The original gel is provided in “Supplementary Information”.

The dNTPs synthesized with the lysate were next tested as the dNTP source for a standard PCR (Fig. 1c). For this, 2.5 μL of the dNTP synthesis reaction mixture was transferred directly to a PCR mixture (25 μL) lacking dNTPs, and DNA amplification from plasmid DNA template was carried out through a standard PCR protocol. As seen in Fig. 3, a DNA amplicon of the expected size (0.9 kb) was observed as a bright single band on an agarose gel. No band was observed in the absence of DNA template or in other appropriate control reactions. This demonstrates that dNTPs synthesized from deoxynucleosides by the recombinant cell lysate may be employed directly as PCR substrates without prior isolation.

**Figure 4:**
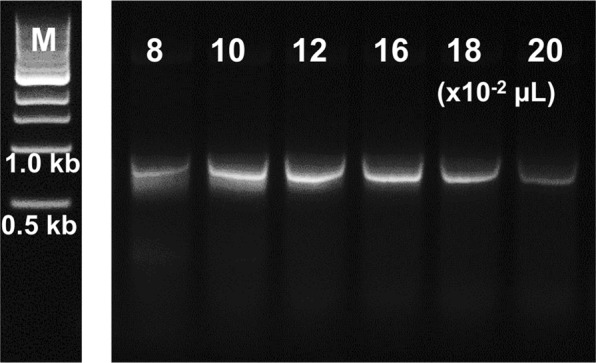
Agarose gel of PCR products with increasing concentrations of dNK lysate and dNTPs synthesized *in situ* from deoxynucleosides (0.2 mM). NEB 1 kb DNA size marker (M) and 0.08–0.2 μL of cell lysate per 25 μL reaction. Expected product size is 0.9 kb. All lanes are from a single gel image, cropped for clarity and conciseness. The original gel is provided in “Supplementary Information”.

Having demonstrated the compatibility of the dNTP synthesis reaction mixture with PCR, a one-pot process where dNTP synthesis and PCR occur sequentially was considered. This circumvents the requirement for separate handling steps, further streamlining the process and reducing the risk of cross-contamination^33^. To test this, the components required for dNTP synthesis were included in the PCR mixture (in place of dNTPs and without prior incubation), and a 37 °C incubation to allow dNTP synthesis was added to the beginning of the thermal cycling program. DNA product was again obtained for a range of tested lysate concentrations, as shown in Fig. 4 (also Supplementary Information, Fig. S5). When the lysate concentration was increased beyond a certain point, less DNA amplification was apparent. This effect is probably due to the accompanying increase in the concentration of background degradative enzymes. In line with our previous observations for ATP dependent reactions catalyzed by cell lysates^14^, these effects would probably be alleviated through adjustment of other reaction parameters (e.g., exchange of buffer, higher substrate concentrations). Nonetheless, this result clearly demonstrates that the two processes of dNTP synthesis and PCR are compatible and may be combined, with no observable inhibition at practical lysate concentrations (~0.1 μL per 25 μL reaction). Control reactions lacking deoxynucleosides, ATP, acetyl phosphate, template DNA, or cell lysate did not result in DNA synthesis, confirming that the origin of the dNTPs for the PCR is the cell-lysate catalyzed reaction shown in Fig. 1a.

**Figure 5:**
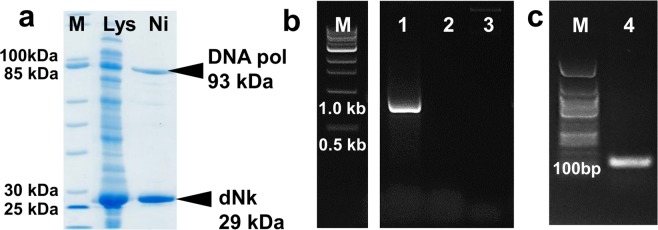
Analysis and application of recombinant lysate from the co-expression of deoxynucleoside kinase (dNK) and DNA polymerase (DNA pol). (**a**) SDS-PAGE analysis of lysate from *E. coli* BL21 star(DE3) overexpressing dNK and DNA polymerase (Lys), Ni-affinity purifed lysate fraction (Ni), and molecular weight marker (M) (NEB Broad Range 10–200 KDa). (**b**) Agarose gel showing NEB 1 kb DNA size marker (M), PCR with dNTPs synthesized *in situ* from deoxynucleosides (1), control PCR without deoxynucleosides (2), and control PCR without lysate (3). Expected product size is 0.9 kb. (**c**) Agarose gel showing NEB 50 bp DNA size marker (M), and PCR with 0.1 kb DNA target (4). All lanes in each panel are from a single gel image, cropped for clarity and conciseness. The original gels are provided in “Supplementary Information”.

Though this streamlined protocol omits adding dNTPs, DNA synthesis still requires use of an added DNA polymerase. This enzyme is typically prepared through recombinant overexpression^3^ and so, encouraged by the compatibility of our cell-free biosyntheses, we considered recombinantly incorporating this activity into the lysate. Consolidation of nucleotide and polymerase production processes would further improve PCR reagent affordability and accessibility. In the event, we chose the DNA polymerase from *Pyrococcus furiosus* due to its low error-rate and utility in molecular biology and diagnostics^17^. The gene present within pET16b.Pfu (gifted by Sung-Hou Kim)^18^ was subcloned with an N-(His)_6_-tag into the pACYC-Duet expression vector. As pACYC and pETMCSIII (used to express dNK) have compatible origins and different antibiotic resistance, co-expression of the dNK and DNA polymerase could be evaluated. Initial screens showed that *E. coli* BL21 star(DE3) resulted in the best combined overexpression, so this strain was employed instead of BL21(DE3) for subsequent work. SDS-PAGE of the crude lysate and the Ni(II)-affinity-purified fraction prepared from cells grown in LB (1 L) and in the presence of ampicillin and chloramphenicol showed two overexpressed N-(His)_6_-tagged recombinant proteins with the migrations expected for dNK and DNA polymerase (Fig. 5a).

To test whether the lysate contained both recombinant activities while retaining the necessary endogenous kinases, the one-pot dNTP synthesis and PCR was carried out as above but without commercial polymerase or dNTPs. Yet again, the expected DNA amplicon was observed as a bright single band on an agarose gel (Fig. 5b). Furthermore, comparison of PCRs with and without initial supplementation with 0.2 mM of each of the commercial dNTPs, which would be predicted to increase the total dNTP concentrations by at least 2–4 fold (based on the synthetic yields under more concentrated conditions - see Fig. 4), showed only a 1.6-fold increase in DNA amplicon, as determined through the relative intensity of gel DNA bands (see Supplementary Information, Fig. S4). Similar increases in the total dNTP concentrations would be expected to be achievable without dNTP supplementation, by instead using higher concentrations of the deoxynucleosides, more lysate and/or longer synthesis times. All the above observations confirm that the hyperthermophilic DNA polymerase is active and able to effectively utilize dNTPs produced from deoxynucleosides *in situ*. In this way, commercial dNTPs and polymerase are replaced by an affordable mixture of reagents, buffer and lysate, that is easy to prepare and may be prepared in advance. The same protocol was also applied for the production of a smaller DNA amplicon (0.1 kb) without issue, showing that the system is not limited to larger products (Fig. 5c). The co-expression lysate is highly active and may be employed at very low concentrations (approx. 0.015 μL per 25 μL PCR). This means that lysate from just 1 L of bacterial growth supports ~10^6^ PCRs representing remarkable reduction in cost per test even at lab scale.

**Figure 6:**
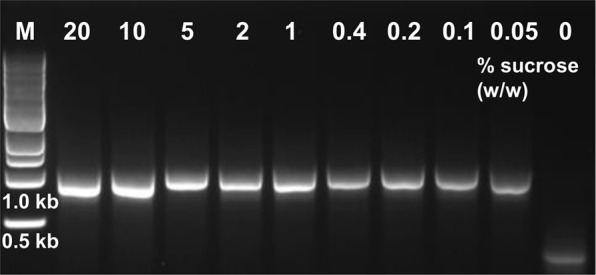
Agarose gel of PCRs with lyophilized lysate. NEB 1 kb DNA size marker (M), and using lysate lyophilized in the presence of 20–0.1% w/w sucrose lyoprotectant and in its absence. Expected product size is 0.9 kb. All lanes are from a single gel image, cropped for clarity and conciseness. The original gel is provided in “Supplementary Information”.

The above procedures were developed with ATP as the only added triphosphate, and then in sub-stoichiometric amounts. Nonetheless, we investigated the possibility of replacing it altogether with ADP or AMP. We previously found both to be suitable replacements in lysate-catalyzed biosyntheses, as they are rapidly converted by endogenous kinases to ATP with acetyl phosphate^14,26,28,29^. Indeed, when either was applied to the lysate PCR in place of ATP, uninhibited DNA synthesis was observed (Supplementary Information, Fig. S2). Cell extracts typically require cold storage, however lyophilized extracts retain cell-free protein synthesis capacity at room temperature for months^20^. Evaluating this protocol with our lysate for PCR, when lyophilized with sucrose as a lyoprotectant, the lyophilized material also retained DNA synthesis activity following rehydration (Fig. 6; also Supplementary Information, Fig. S6). Furthermore, by freeze-drying the requisite amount of lysate and acetyl phosphate into individual wells, PCR could be carried out after a simple one-step reconstitution using a stable buffer-reagent mix, further reducing process complexity (Supplementary Information, Fig. S3).

In conclusion, a single recombinant cell lysate of *E. coli* catalyzes the transformation of unphosphorylated deoxynucleosides into dNTPs coupled to *in situ* DNA polymerization. In this way PCR is carried out without the limitations associated with costly and labile dNTPs and polymerases. The reagents used here are stable and inexpensive, while enough cell lysate to carry out ~10^6^ PCRs is generated from just 1 L of bacterial culture. It is therefore envisaged that an inexpensive lysate-based mixture could be produced in advance and delivered to remote and low-tech settings, for application through a simplified protocol without costly reagents. This work demonstrates the utility of lysate-based biosynthesis, as the reported cascade entails fourteen enzymatic steps followed by a series of polymerase-catalyzed DNA elongation reactions. It also highlights the potential and versatility of cell-free synthetic biology in diagnostics^34^. Cell lysates have been employed in diagnostics to carry out cell-free synthesis of reporter proteins in response to nucleic acid (RNA) amplified using a commercial kit^8^. We show that NAA is not only compatible with cell-free lysate, but may be supported by the lysate itself. Overall, this approach holds great promise for affordable and simplified application by non-skilled personnel without intensive laboratory support, and we are currently working on application to real-world diagnostics.

## Methods

### General methods

Chemicals reagents were from Sigma-Merck. Deoxynucleosides, dNMPs, dNTPs, AMP, ADP and ATP were all of >99% purity. Acetic anhydride and H_3_PO_4_ (85%) used for the synthesis of acetyl phosphate were ACS grade. Taq DNA polymerase and broad range (10–200 kDa) molecular weight markers were from New England Biolabs (MA, USA). SDS-PAGE gels (15%) were run on a Mini-protein Tetra system and stained with Bio-safe Coomassie Blue (Biorad). His-tagged proteins were purified with His-GraviTrap or His-SpinTrap kits from GE Healthcare.

### Plasmid construction

The DNA sequence for *D. melanogaster* dNK (Uniprot P61875) was commercially synthesized (GeneArt, Germany) codon optimized for *E. coli* and shipped in the pMK plasmid, flanked by the NdeI and EcoRI restriction sites, which were used to sub-clone the gene into pETMSCIII^32^ for expression with a N-(His)_6_-tag with ampicillin resistance. Plasmid pET16b.Pfu (Addgene #12509) containing *Pyrococcus furiosus* N-(His)_6_-DNA polymerase was gifted by Sung-Hou Kim^18^. The coding region was PCR amplified, flanked by NcoI and SalI restriction sites, which were used to sub-clone the gene into pACYCDuet (Merck, Germany) with chloramphenicol resistance and a compatible origin of replication with pETMCSIII. Coding region integrities were confirmed by Sanger sequencing (ARBF, ANU JCSMR).

### Preparation of lysate

Lysogeny broth (LB) (50 mL) supplemented with 50 mg mL^−1^ ampicillin was inoculated with *E. coli* BL21(DE3) transformed with pETMCSIII-dNK. Cells were grown overnight (~18 h, 37 °C, 180 rpm) before harvesting by centrifugation (4000 × *g*, 15 min). Cell pellets were resuspended in 5 mL Tris-acetate buffer (10 mM, pH 8.3) containing 16 mM KOAc, 14 mM MgOAc, 1 mM phenylmethylsulphonyl fluoride, and 1 mM dithiothreitol. Lysis was performed over ice by sonication with an Omni Sonic Ruptor 400 equipped with an ORT-375 processing tip (50% power, 50% pulse, 6 min). Lysate was clarified by centrifugation (20,000 × *g*, 1 h), snap frozen and stored at −20 °C. Lysates prepared in this way may be stored without issue, for use over several months.

### Preparation of lysate with overexpressed dNK and DNA polymerase

Lysogeny broth (1 L) supplemented with 50 mg mL^−1^ ampicillin and 25 mg mL^−1^ chloramphenicol was inoculated with a starter culture (10 mL) of *E. coli* BL21 star(DE3) co-transformed with pETMCSIII-dNK and pACYCDuet-Pfu. This strain of *E. coli* is identical to BL21(DE3), except for the deletion of RNase E which enhances mRNA half-lives, and was found to lead to improved protein co-expression yields and therefore was employed here. In accordance with the co-expression method of Tolia *et al*.^35^, cells were incubated (37 °C, 180 rpm) until OD_600_ 0.3, then the temperature was reduced to 30 °C. Expression was induced at OD_600_ 0.6 with ITPG (1 mM) and incubation continued (30 °C,180 rpm) for 14 h. Cells were harvested by centrifugation (4000 × *g*, 15 min) and stored at −80 °C until further processing. Cell pellets were thawed on ice and resuspended (1 mL g^−1^ wet cells) in Tris-acetate buffer (10 mM, pH 8.3) containing 16 mM KOAc, 14 mM MgOAc, 1 mM phenylmethylsulphonyl fluoride, and 1 mM dithiothreitol before sonication, clarification and snap freezing as above.

### Synthesis of dNTPs from deoxynucleosides and HPLC analysis

Synthesis of dNTPs was carried out in HEPES buffer (pH 7.4, 200 mM) with dAdo, dCyt, dGuo and dThy (1 mM each), ATP (1 mM), MgCl_2_ (10 mM), acetyl phosphate (50 mM) and lysate from dNK overexpression (4 μL per mL). All reactions were initiated through addition of lysate. Acetyl phosphate was prepared as a 1 M aqueous solution from acetic anhydride and H_3_PO_4_ (85%) following a previously described method^14,15^, and used directly in subsequent reactions. Phosphate buffer, used in our previous work on nucleotide synthesis^14,28^, was found to inhibit standard PCR and was therefore replaced by HEPES. The mixture was incubated at 37 °C, then samples at set time-points were quenched and analyzed by HPLC (Fig. 2b).

Deoxynucleosides, deoxynucleotides and adenosine nucleotides were analyzed by HPLC (Agilent 1100) with an Alltima HP C18 column (5 *μ*, 250 × 5.6 mm; 7.5 × 4.6 mm guard), gradient elution (87–70%) with pH 5.0 aqueous ammonium dihydrogen phosphate 60 mM/5 mM tetrabutylammonium phosphate and 5 mM tetrabutylammonium phosphate in MeOH, monitoring at 259 nm (absorption maximum for all nucleobases). Species separation is based on relative charge, due to ion-pairing with tetrabutylammonium in the mobile phase, and hydrophobic interactions with the C18 stationary phase, with a greater number of more negatively charged phosphates resulting in longer retentions. Retention times were determined through comparison with commercial standards and were as follows: dCyt - 3.9 min, dGuo - 4.5 min, dThy - 5.1 min, dAdo - 6.5 min, dCMP - 4.7 min, dGMP - 6.1 min, dTMP - 6.8 min, dAMP – 9.2 min, dCTP – 16.9 min, dGTP – 23.8 min, dTTP – 24.8 min, dATP – 25.6 min, ATP – 25.2 min. For each dNTP, quantification was carried out by comparison with a standard response curve generated with commercial standards, which was linear over the concentrations measured (up to 2 mM).

### PCR with lysate synthesized dNTPs

PCR reactions (25 μL) were performed in NEB Thermopol (20 mM Tris-HCl at pH 8.8 and 25 °C with 10 mM (NH_4_)_2_SO_4_ 10 mM KCl, 2 mM MgSO_4_, 0.1% Triton-X-100), 1 U NEB Taq polymerase, 0.2 μM forward and reverse primers (RSC514, RSC515^32^; Supplementary Information, Table S1), template DNA (25 ng plasmid pETMCSIII-dNK; Supplementary Information, Table S2) and 2.5 μL of the dNTP synthesis reaction mixture to a final volume of 25 μL. The thermal treatment was 95 °C initial denaturation, 3 min; 35 cycles [95 °C denaturation, 15 s; 55 °C annealing, 30 s; 72 °C extension 30–90 s); 72 °C final extension, 7 min; then held at 4 °C until analysis by agarose gel electrophoresis. For *in situ* dNTP synthesis, PCR was carried out as above but with the addition of 40 mM HEPES, 0.2 mM of each deoxynucleoside, 0.2 mM ATP, 10 mM acetyl phosphate and 0.02–0.22 μL lysate from the overexpression of dNK (final PCR mixture pH 8.2) instead of the dNTP synthesis reaction mixture. A 240 min incubation at 37 °C was added to the beginning of the thermal treatment.

### Lysate catalyzed *in situ* dNTP synthesis and PCR

PCRs were carried out in a mixture of HEPES (40 mM) and Tris-HCl (20 mM) at pH 8.2 and 25 °C, with 10 mM KCl, 10 mM (NH_4_)_2_SO_4_ 3 mM MgSO_4_, 0.1% Triton-X-100, 4% DMSO, 0.2 μM forward and reverse primers (RSC514, RSC515), 10 mM acetyl phosphate, 0.2 mM ATP and 0.2 mM of each deoxynucleoside, 25 ng of template DNA and 0.015–0.03 μL lysate from the co-expression of dNK and DNA polymerase. Addition of 4% DMSO improved amplification and was therefore employed here. For the 0.1 kb amplicon, alternate DNA template (pETMCSIII-Ppm; Supplementary Information, Table S2) and primers were used (Supplementary Information, Table S1). The thermal treatment was the same as for the PCRs with *in situ* dNTP synthesis described above (“PCR with Lysate Synthesized dNTPs).

### Agarose gel electrophoretic analysis of PCR products

Size and purity of lysate-catalyzed PCR products were assesed through electrophoretic migration. Electrophoresis of agarose DNA gels was carried out on a wide Mini-sub Cell GT system in TAE buffer with 0.1 × GelRed nucleic acid stain (Biotium, 10,000 × in DMSO) added directly to the gel. For analysis of the 0.9 kb DNA amplicon, 1.2% agarose gels were cast in a 100 × 150 mm format with 40 sample wells and run at 120 V for 20 mins. For the 0.1 kb amplicon, 2.5% agarose gels were cast in the same format and run at 180 V for 15 mins. Analysis samples were prepared by mixing 10 μL of the analyte PCR reaction with 2 μL Gel Loading Dye (Purple 6x, no SDS, New England Biolabs) and gel well-loading of the entire volume. Following electrophoresis, the entire gel was transferred to the Axygen Gel Doc system for imaging of the electrophoresed bands, at 302 nm (2–4 sec exposure) and DNA amplicon migration was compared against suitable molecular weight markers (100 bp or 1.0 kb, both from New England Biolabs) that had been electrophoresed on the same gel.

### Lysate lyophilization

Lysates from the co-expression of dNK and DNA polymerase were lyophilized in the presence of varying concentrations of sucrose, as a lyoprotectant. The lysate (8 μL) was diluted 125-fold with H_2_O to a final volume of 1 mL, and 2.5 μL aliquots were transferred to the bottom of individual PCR reaction wells. These were mixed with an equal volume of sucrose stock solutions in H_2_O of varying concentrations (0–40% w/w), so that the final sucrose concentration in the PCR-well-mixtures ranged from 0–20% w/w. Samples were flash-frozen with liquid N_2_ and loaded onto a PCR-tube-rack, placed in a freeze-drying glass vessel (600 mL, Labconco) and connected to a drying chamber valve port of a Labconco FreeZone 4.5 L Benchtop Freeze Dry system set to −51 °C, 0.1 mbar, and lyophilized for 6 h. For DNA amplification, lyophilized lysate was rehydrated with a buffer mixture containing HEPES (40 mM), Tris-HCl (20 mM) pH 8.2, with 10 mM KCl, 10 mM (NH_4_)_2_SO_4_ 3 mM MgSO4, 0.1% Triton-X-100, 10 mM acetyl phosphate, 0.2 μM forward and reverse primers (RSC514, RSC515), 0.2 mM of each deoxynucleoside and ATP, followed by addition of template DNA, and PCR was carried out as before (see above “Lysate Catalyzed *in situ* dNTP Synthesis and PCR**”**). Similar results were obtained when the necessary acetyl phosphate was co-lyophilized with the lysate and withheld from the buffer mixture (Supplementary Information, Fig. S3).

## Supplementary information

Supplementary information
