# Supplementary material for: DNA amplification with in situ nucleoside to dNTP synthesis, using a single recombinant cell lysate of E. coli
Source: Sci Rep. 2019 Oct 30;9:15621. doi: 10.1038/s41598-019-51917-z (PMC6821818; doi:10.1038/s41598-019-51917-z)

Supplementary Information for

DNA amplification with *in situ* nucleoside to dNTP synthesis, using a single recombinant cell lysate of *E. coli*

Thomas D. Loan,[1] Christopher J. Easton,[1] Apostolos Alissandratos*[,1,2]

[1] Research School of Chemistry, Australian National University, Canberra, Australia.

[2] CSIRO Synthetic Biology Future Science Platform, Australian National University, Canberra, Australia.

*Apostolos.Alissandratos@anu.edu.au

1. Activity assay of purified deoxynucleoside kinase (dNK)

dNK activity assays were carried out in 0.5 mL Tris (pH 7.4, 50 mM), ATP (1 mM), $MgCl_2$ (5 mM) and dAdo, dCyt, dGuo or dThy (1 mM). Reactions were incubated at 37 °C and samples were withdrawn at defined time intervals, quenched in an equal volume of 0.02% SDS then directly analyzed by HPLC (see "Synthesis of dNTPs from Deoxynucleosides and HPLC Analysis" in Methods section) as shown in Figure S1.

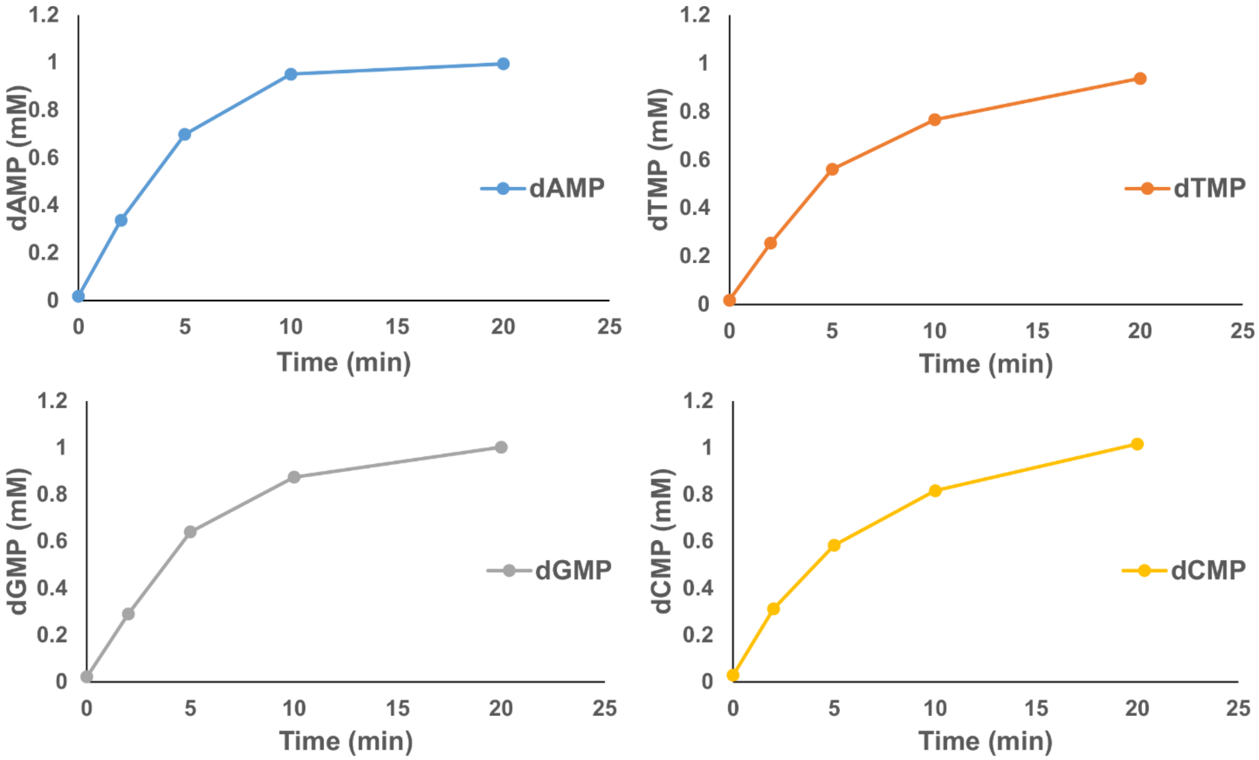

Figure S1: Reaction progress for dNMP synthesis from deoxynucleosides (1 mM) catalyzed by Ni(II)-affinity-purified dNK.

2. PCR with AMP/ADP in place of ATP.

The effectiveness of substituting ATP for ADP or AMP was assessed by carrying out the PCR with 0.2 mM of each compound in place of ATP. These reactions were carried out in HEPES (40 mM), Tris-HCl (20 mM) at pH 8.2 at 25°C, with 10 mM KCl, 10 mM $(NH_4)_2SO_4$ 3 mM MgSO4, 0.1 % trition-X-100 0.2 µM forward and reverse primers (RSC514, RSC515), 0.2 mM of each deoxynucleoside, 10 mM acetyl phosphate and 25 ng of template DNA (pETMCSIII-dNk) with 0.2 mM of ATP, ADP or AMP. Thermal cycle was 95°C initial denaturation, 3 min; 35 cycles [95°C denaturation, 15 s; 55°C annealing, 30 s; 72°C extension 30-90 s) and 72°C final extension, 7 min; held at 4°C until analysis by agarose gel electrophoresis (Figure S2).

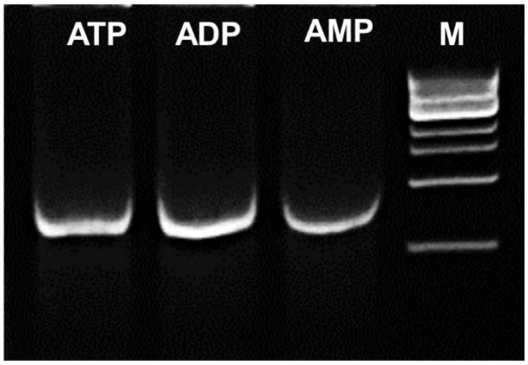

Figure S2: Agarose gel of lysate catalyzed PCR amplicons using ATP, ADP or AMP. All showed comparable amplicons. DNA size marker is 1 kb NEB (M).

3. Lysate and acetyl phosphate co-lyophilization and application for PCR

Lysate from the co-expression of dNk and DNA polymerase was diluted 125-fold with an aqueous solution of acetyl phosphate (100 mM concentration in mixture). This was then aliquoted (2.5 μL) to individual PCR reaction wells and mixed with an equal volume of 20% w/w sucrose solution, to produce a 5 μL mixture containing 10% w/w sucrose, 50 mM acetyl phosphate, 0.02 μL lysate. These were flash frozen in liquid N_2 and lyophilized for 6 h at -51 °C, ~0.1 mBar using a FreeZone 4.5 L benchtop freeze dryer (Labconco, MO).

For DNA amplification, lyophilized lysate in individual wells was rehydrated with a buffer mixture containing HEPES (40 mM), Tris-HCl (20 mM) pH 8.2, with 10 mM KCl, 10 mM $(NH_4)_2SO_4$ 3 mM MgSO4, 0.1 % Triton-X-100, 0.2 μM forward and reverse primers (RSC514, RSC515), 0.2 mM of each deoxynucleoside and ATP, followed by addition of template DNA and PCR was carried out as before. Analysis by gel electrophoresis showed the desired DNA amplicon (Figure S3).

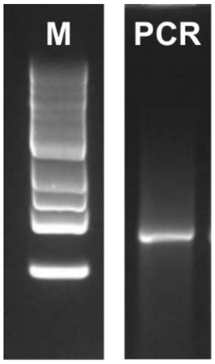

Figure S3: Agarose gel of lysate freeze dried with acetyl phosphate and 0.5 mg sucrose (20% w/v) in a PCR tube and reconstituted with buffer and reagent mixture before dNTP synthesis and PCR. Marker is 1 kb NEB (M). Lanes are from a single gel image, cropped for clarity and conciseness with no further modification. The original gel is provided in “Supplementary Information”.

4. Comparison of PCRs with and without supplementation with commercial dNTPs

A PCR with *in situ* generation of dNTPs from deoxynucleosides (0.2 mM) was compared to a PCR containing the same mixture, but additionally supplemented with commercial dNTPs (0.2 mM; as indicated for commercial PCR and equal to the starting concentration of deoxynucleosides). The reactions were carried out in HEPES (40 mM) and Tris-HCl (20 mM) at pH 8.2 and 25°C, with 10 mM KCl, 10 mM $(NH_4)_2SO_4$ 3 mM $MgSO_4$, 0.1% Triton-X-100, 4% DMSO, 0.2 μM forward and reverse primers (RSC514, RSC515), 10 mM acetyl phosphate, 25 ng of template DNA, 0.03 μL lysate from the co-expression of dNK and DNA polymerase, 0.2 mM ATP and 0.2 mM of each deoxynucleoside, and with 0.2 mM of each of the dNTPs for the supplemeted reaction. PCR was then performed as before (Methods section “Lysate Catalyzed *In Situ* dNTP Synthesis and PCR”), followed by agarose gel electrophoresis (Figure S4). The gel image containing both PCR products was analyzed using Image Studio Lite Ver 5.2 (LICOR). Both lanes were imported in a single image and DNA bands were defined through the auto-adjust feature, for determination of signal intensity (Figure S4). The relative DNA band intensities for the PCR using deoxynucleosides only and the PCR supplemented with commercial dNTP was 1.0:1.6 (20900000:33700000).

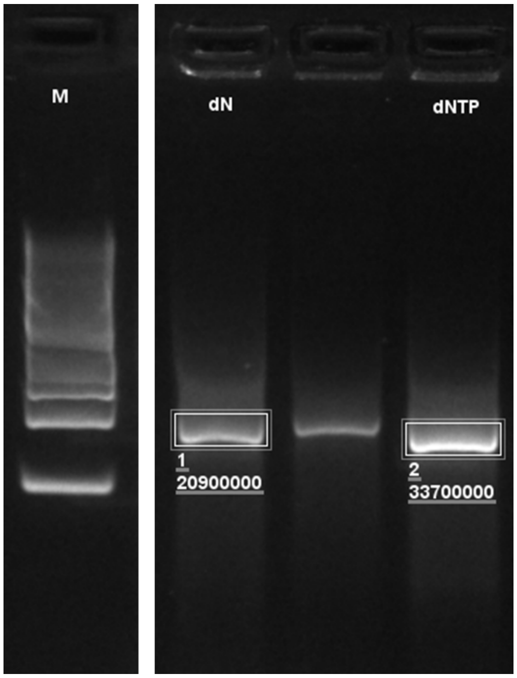

Figure S4: Agarose gel of PCR amplicons using 0.2 mM deoxynucleosides for *in situ* dNTP generation (dN), further supplemented with 0.2 mM commercial dNTPs (dNTP). The DNA size marker is 1 kb NEB (M). Band intensity is raw output from Image Studio Lite Ver 5.3 for auto-adjusted DNA band selection. Lanes are from a single gel image, cropped for clarity and conciseness with no further modification. The original gel is provided in “Supplementary Information”.

5. Gels with relative DNA band intensities

Gels presented in Figures 4 and 6 in the main manuscript are shown here (Figures S5 and S6) with relative DNA band intensities measured through the use of Image Studio Lite V5.2 (LICOR), as described above in "Comparison of PCRs with and without supplementation with commercial dNTPs" (page S5), and calculated as a percentage of the signal for the band with the highest intensity.

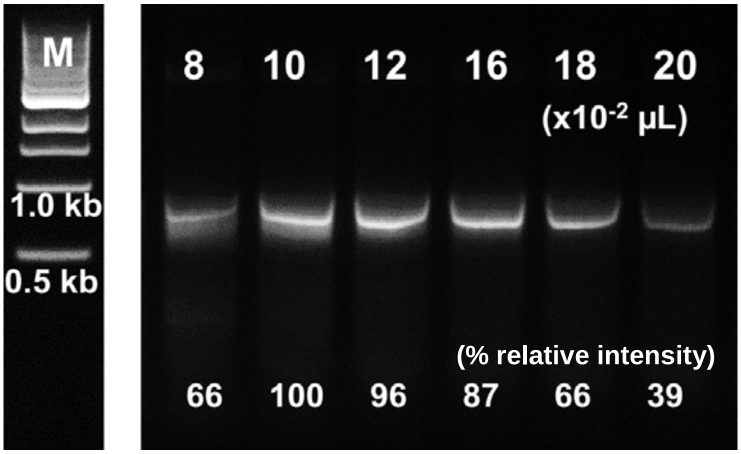

Figure S5: Agarose gel of PCR products with increasing concentrations of dNK lysate and dNTPs synthesized *in situ* from deoxynucleosides (0.2 mM). NEB 1kb DNA size marker (M) and 0.08 – 0.2 μL of cell lysate per 25 μL reaction. Expected product size is 0.9 kb. DNA band intensities are presented as a percentage of signal for the band with the highest intensity (Image Studio Lite V5.2). All lanes are from a single gel image, cropped for clarity and conciseness. The original gel is provided in "Supplementary Information".

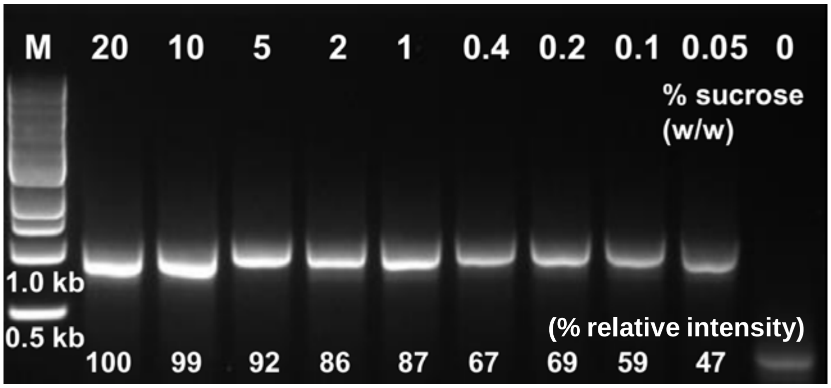

Figure S6: Agarose gel of PCRs with lyophilized lysate. NEB 1 kb DNA size marker (M), and using lysate lyophilized in the presence of 20 – 0.1 % w/w sucrose lyoprotectant and in its absence. Expected product size is 0.9 kb. DNA band intensities are presented as a percentage of the signal for the band with the highest intensity (Image Studio Lite V5.2). All lanes are from a single gel image, cropped for clarity and conciseness. The original gel is provided in "Supplementary Information".

6. Sequences of oligonucleotide primers and plasmids

Table S1. Oligononucleotide primers employed in PCR described.

Primer	Sequence	Origin
RSC514	5’-cgactcactatagggagaccacaac - 3’	Neylon et al.[1]
RSC515	5’-cctttcgggctttgttagcag - 3’	Neylon et al.[1]
PpmF2	5’- cgtgaacacattccggtact - 3’	This study
PpmR2	5’- ttgccagagtctgaccaatatc - 3’	This study

Table S2. Plasmids employed in this study.

Plasmid	Insert	Origin
pETMCSIII-dNk	dNk (Uniprot: Q9XZT6)	This study
pACYC-Pfu	Pfu DNA polymerase (Uniprot: P61875)	This study
pETMCSIII-Ppm	*deoB* gene sequence (Uniprot: A0A140NEF7)	Loan et al.[2]

Suplementary Information References

(1) Neylon, C.*et al*. Interaction of the *Escherichia coli* replication terminator protein (Tus) with DNA: A model derived from DNA-binding studies of mutant proteins by surface plasmon resonance, *Biochemistry* **39**, 11989-11999 (2000)
(2) Loan, T. D., Easton, C. J. & Alissandratos, A. Recombinant cell-lysate-catalysed synthesis of uridine-5'-triphosphate from nucleobase and ribose, and without addition of ATP, *New Biotechnol.* **49**, 104-111 (2019)

Supplementary Gel Images for

DNA amplification with *in situ* nucleoside to dNTP synthesis, using a single recombinant cell lysate of *E. coli*

Thomas D. Loan,[1] Christopher J. Easton,[1] Apostolos Alissandratos*,[1,2]

[1] Research School of Chemistry, Australian National University, Canberra, Australia.

[2] CSIRO Synthetic Biology Future Science Platform, Australian National University, Canberra, Australia.

*Apostolos.Alissandratos@anu.edu.au

Areas of interest are highlighted by a white square.
Labels correspond to those in the main paper and “Supplementary Information”.

Figure 3

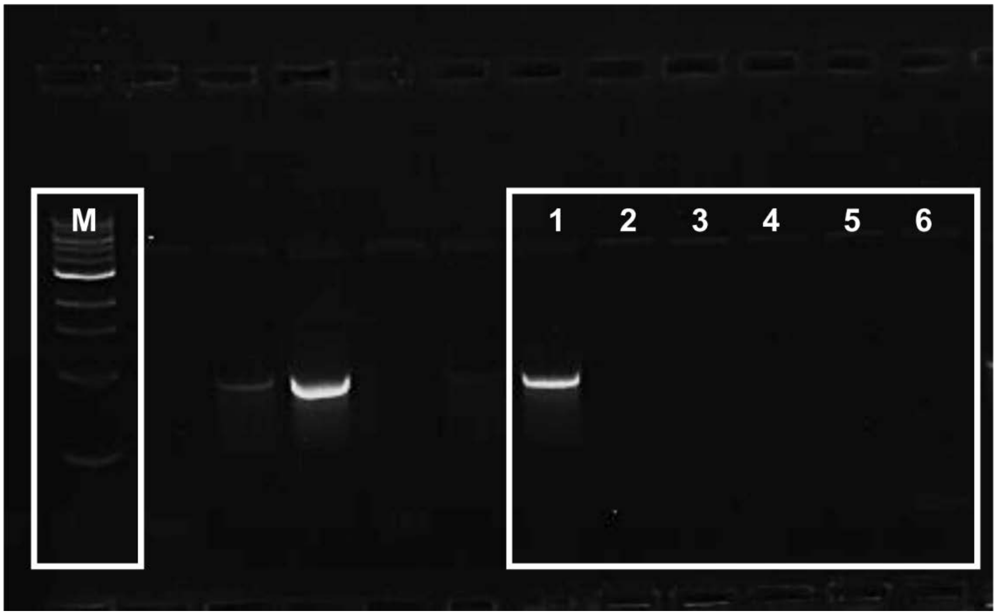

Figure 4 and Figure S5

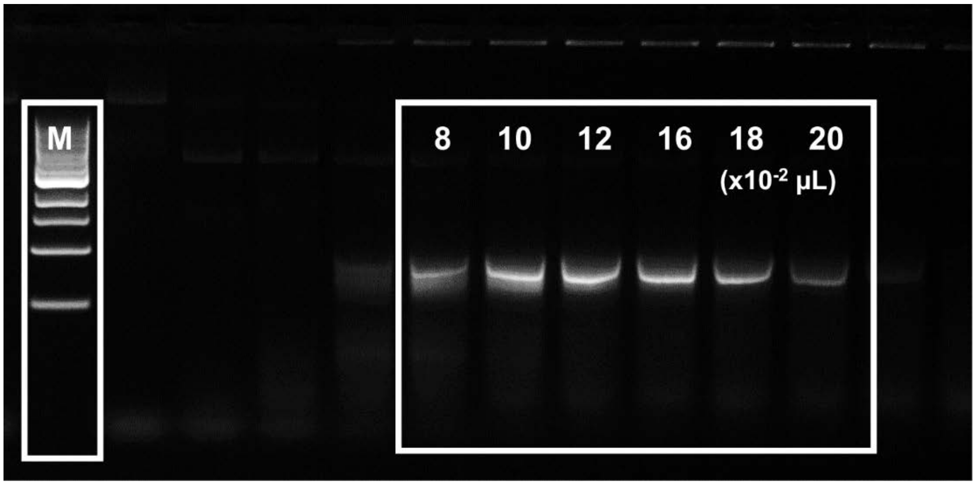

Figure 5b

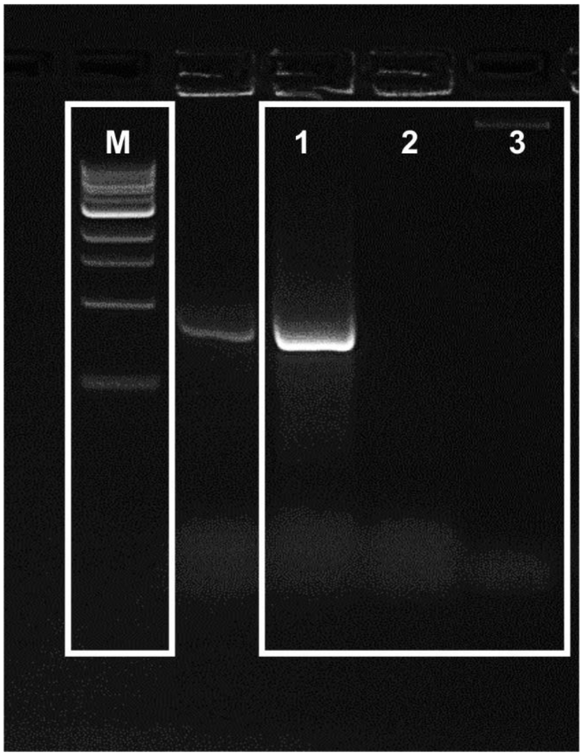

Figure 5c

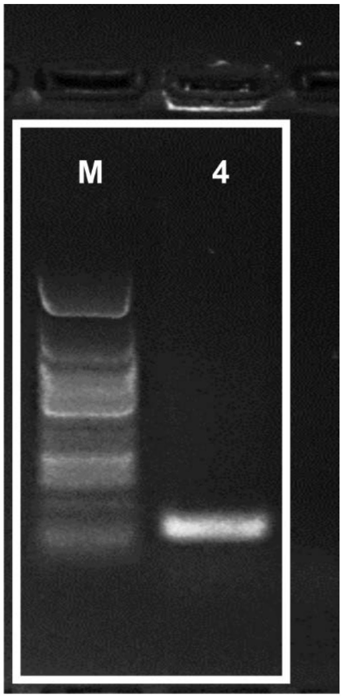

Figure 6 and Figure S6

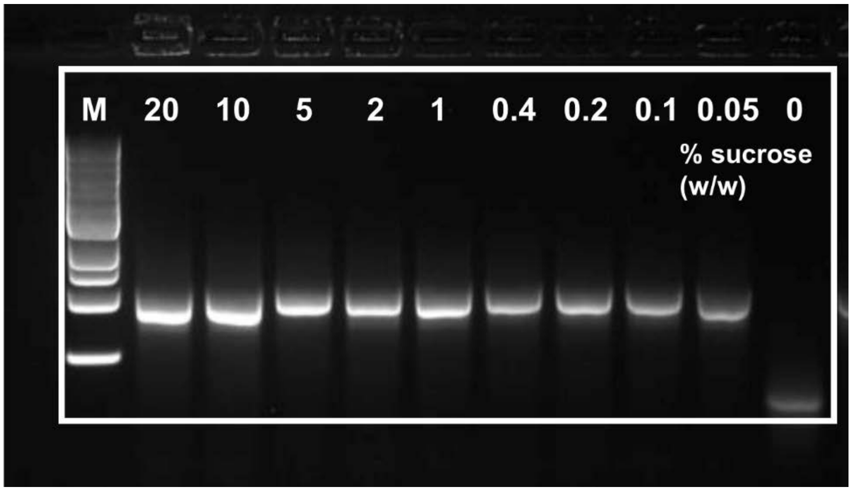

Figure S3

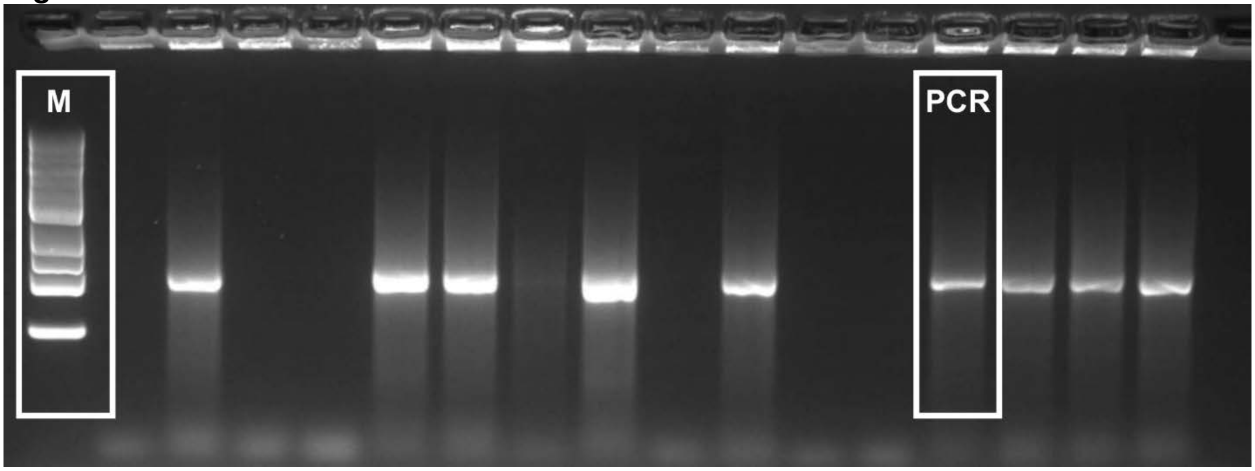

Figure S4

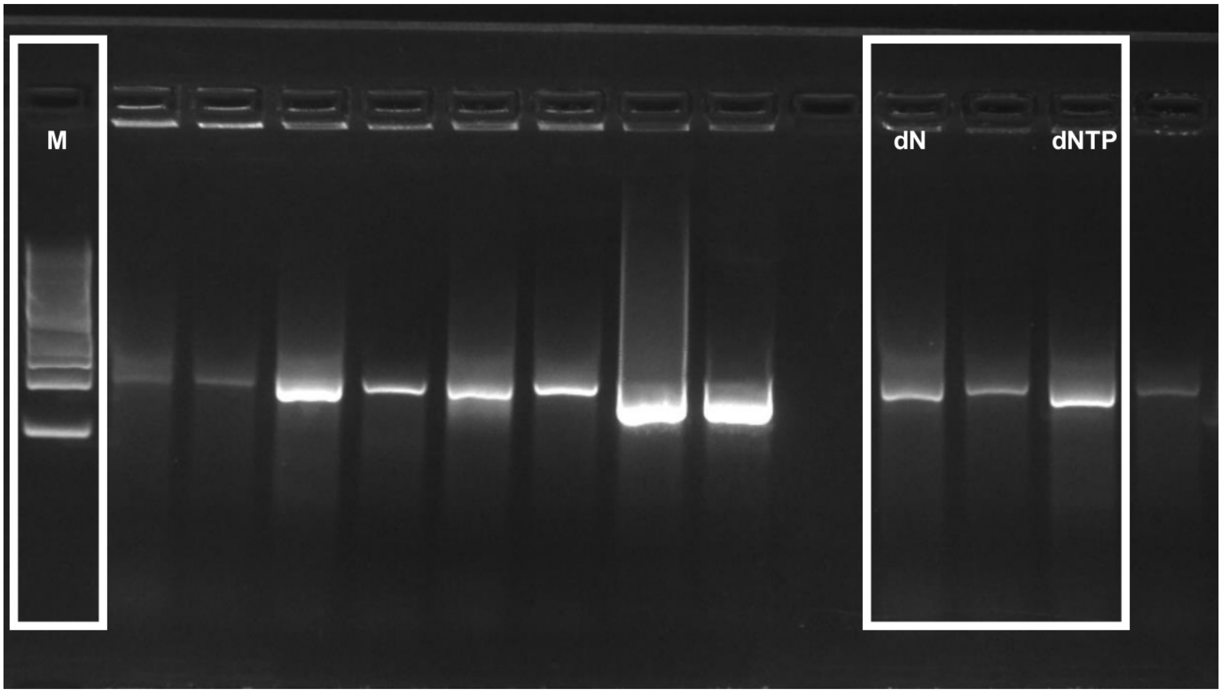

Supplement: Supplementary file 1 — Supplementary information [file 41598_2019_51917_MOESM1_ESM.pdf]
